# Coexisting with sharks: a novel, socially acceptable and non-lethal shark mitigation approach

**DOI:** 10.1038/s41598-020-74270-y

**Published:** 2020-10-15

**Authors:** Kye R. Adams, Leah Gibbs, Nathan A. Knott, Allison Broad, Martin Hing, Matthew D. Taylor, Andrew R. Davis

**Affiliations:** 1 School of Earth, Atmospheric and Life Sciences, University of Wollongong, Northfields Avenue, Wollongong, NSW 2522 Australia; 2 School of Geography and Sustainable Communities, University of Wollongong, Northfields Avenue, Wollongong, NSW 2522 Australia; 3 School of Environmental and Life Sciences, University of Newcastle, University Drive, Callaghan, NSW 2308 Australia

**Keywords:** Environmental impact, Psychology and behaviour, Sustainability, Human behaviour, Conservation biology

## Abstract

Conflict between humans and large predators is a longstanding challenge that can present negative consequences for humans and wildlife. Sharks have a global distribution and are considered to pose a potential threat to humans; concurrently many shark species are themselves threatened. Developing strategies for coexistence between humans and this keystone group is imperative. We assess blimp surveillance as a technique to simply and effectively reduce shark encounters at ocean beaches and determine the social acceptance of this technique as compared to an established mitigation strategy—shark meshing. We demonstrate the suitability of blimps for risk mitigation, with detection probabilities of shark analogues by professional lifeguards of 0.93 in ideal swimming conditions. Social surveys indicate strong social acceptance of blimps and preference for non-lethal shark mitigation. We show that continuous aerial surveillance can provide a measurable reduction in risk from sharks, improving beach safety and facilitating coexistence between people and wildlife.

## Introduction

“*Man is not made for defeat*”

— Ernest Hemingway, The Old Man and the Sea

Human–wildlife conflict is a persistent and divisive issue that often results in social and environmental impacts. People can lose their sense of safety, livelihoods, and on rare occasions their lives^1,2^. Consequently, animals are targeted for destruction despite at times being threatened, as has been the case for the white shark *Carcharodon carcharias*^3^ and an array of other apex predators^4^. Human societies often seek to exclude predatory species perceived as threatening to human life, including lions, bears, wolves, crocodiles and sharks, despite their roles as keystone species that regulate ecological processes and maintain biodiversity^5^. Sharks inhabit all oceans and often frequent coastal areas that people use for recreation and work. The wide-ranging distribution of sharks and increasing presence of humans on coastlines creates potential for conflict between people and sharks and presents practical constraints for management.

Although the likelihood of being bitten by a shark when entering the ocean is extremely small, human perception of the overall risk is skewed by the potential horrifying consequences of a shark bite. Shark–human interactions are also disproportionately reported in the media compared to other injuries and deaths (e.g. car accident, disease, violence), which likely contributes to an elevated perception of danger^6,7^. Nevertheless, shark incidents are reported to be occurring more frequently^3,8–10^, driven in most affected areas by rapidly growing human populations and recreational usage of the ocean. Developing strategies to support human and shark coexistence is necessary for conservation of sharks, given the dominance of anthropogenic activities in coastal areas and declines in shark populations^11^. Further complication emerges because the species of shark that bite humans tend to be species that are themselves threatened by humans^11–13^. Considering the dire state of some shark populations globally, strategies to protect ocean users while conserving shark species are necessary to achieve sustainable socio-ecological systems in which these apex predators can exist and fulfil their ecosystem function^14^.

“*Fish," he said, "I love you and respect you very much. But I will kill you dead before this day ends.*”

— Ernest Hemingway, The Old Man and the Sea

Globally, coastal areas are recreation and tourism hotspots. Managing shark–human interactions in these areas can be challenging, requiring management strategies that consider environmental, social and economic outcomes^6^, and balance trade-offs between strategies. A range of strategies exists to mitigate the perceived threat to people, which vary in their impacts on sharks and economic cost. Lethal strategies involve killing sharks and have been employed around the world, including in Australia since the 1930s^1,3,6,13^ and South Africa since the 1950s^16^. Culling programs have been trialled in other areas following shark incidents, including in Hawai ‘i^17,18^, New Zealand and Mexico^6,19^, but were terminated given unacceptable environmental, social and/or economic costs. Lethal strategies most commonly include fishing methods such as gill nets (often referred to as shark netting or shark meshing in this context), longlines, and drum-lines, in which captured sharks of target species are destroyed or tagged and relocated. Such measures may decrease the perceived level of risk^3^, but their effectiveness has been questioned or debated^20^. They are environmentally ‘costly’ in terms of destruction of both target and non-target species, with by-catch to target ratios often in the order of 9:1 for gill netting^8,14^. Due to growing public awareness of the impacts of existing techniques^13,21^, non-lethal methods are increasingly being proposed as sustainable and socially acceptable strategies for reducing actual and perceived risk for ocean users.

Declining public support for those traditional, lethal methods of shark hazard mitigation has given rise to the recent popularity of modern, non-lethal technologies for shark mitigation, detection and deterrence^22^. As with traditional methods, these modern techniques are not 100% effective, and each has its limitations. Surveillance-based techniques involve continuous or intermittent observations (direct or electronic) or detections of sharks, usually with subsequent alerts and action plans after a shark is sighted^3,23,24^. While these methods may provide a non-lethal management alternative to established lethal programs, they have limitations that reduce their global applicability and acceptance^3^. For example, the South African Shark Spotters program relies on spotters using binoculars positioned on high-elevation terrain adjacent to beaches to observe sharks^24,25^. Aerial patrols using helicopters, although used more widely, are costly^26^, provide only short-term coverage with relatively low shark spotting rates (below 20%) and questionable effectiveness^23^. Drones are an emerging technique that have been shown to be effective for monitoring large marine fauna off coastal beaches^26^. Other systems rely on a combination of shark tagging and subsequent detection on acoustic listening stations, and others on the ability of sonar arrays to determine a target shark has been observed and an alert emitted locally and broadcast via web and social media platforms^22^. Social acceptance is key to the success of any approach and there is emerging social support for non-lethal detection and surveillance technologies for shark hazard mitigation^22,26^. Innovative solutions are therefore required to minimise environmental impact and maximise risk reduction. An ideal solution would provide positive coexistence and conservation outcomes, ensuring sustainable shark populations, while quantifying levels of risk and social acceptance^28^.

**Figure 1:**
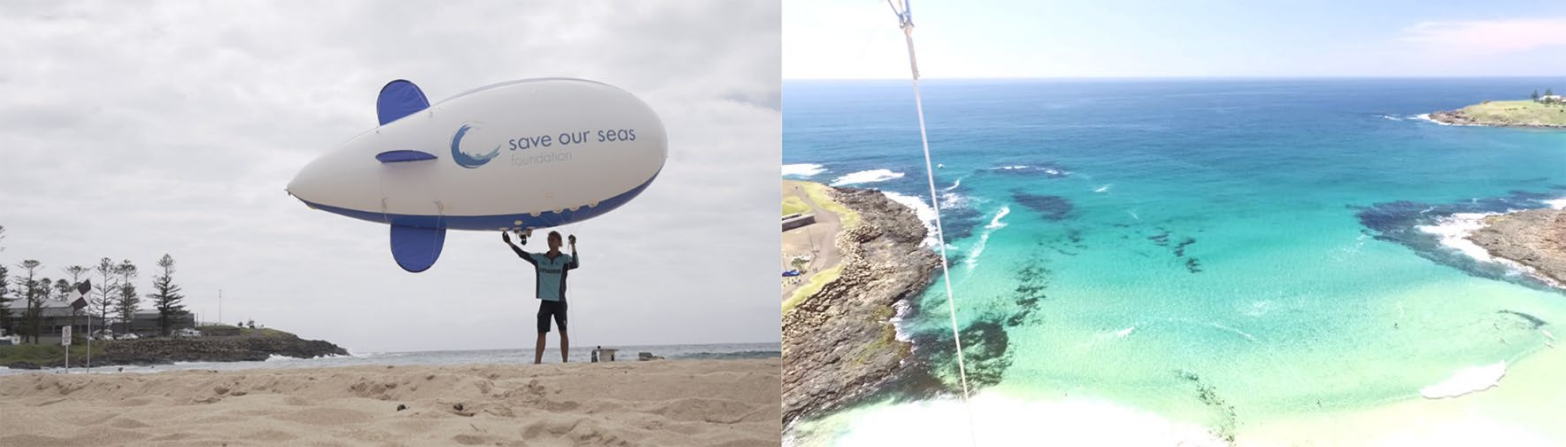
The blimp with camera module attached (left) and the view of Surf Beach from the blimp deployed at 70 m height.

Here, we trial a novel blimp-mounted camera system and assess its effectiveness as a tool to assist lifeguards in detecting sharks at beaches (Fig. 1). We measure the level of risk-reduction achieved by this technique and determine the public acceptance of this novel, non-lethal approach. Our system employs relatively simple and low-cost technology harking back to the Golden Age of Flight: airships, commonly known as blimps. Historically, long flight times and relatively low operating costs resulted in the extensive use of airships for military surveillance and patrol, as well as intercontinental passenger transport^29^. Due to their simplicity and cost-effectiveness in providing a high vantage point and accessing the atmosphere, balloons have also been used for geographical and atmospheric research^30–33^, typically carrying sensors or cameras. These lighter-than-air platforms can provide a stable vantage point for a camera with minimal power consumption; using helium for lift, as opposed to rotors, extends the battery run-time from 30 min (typical of UAV-mounted cameras) to over eight hours^34^. Blimps overcome some of the short-comings of other aerial surveillance techniques including drones, which are restricted by short flight times and potential safety concerns in some populous locations^34,35^. Furthermore, blimps share some of the key advantages of rotary drones: they provide data of high spatial and temporal resolution that are systematic and permanent, along with relatively low operational costs^32^. An additional advantage of using cameras for surveillance is that they provide potential for automated shark detection via algorithms^37,38^. Blimps are also silent, easily deployed and safe in winds up to 40 km/hr with minimal personnel training. Using blimps for continuous beach surveillance could also extend the ability of lifeguards to maintain beach safety by providing an extra vantage point from which swimmers could be observed.

All techniques used for spotting fauna at sea vary in their effectiveness due to sightability errors^26^. These errors are caused by external factor biases (availability bias), and biases introduced by observers (perception bias)^26^ which need to be quantified for any survey technique, particularly one designed for public safety. First, we used the performance of professional lifeguards spotting mobile shark analogues (perception bias) across different meteorological conditions and water depths (availability bias) to assess the effectiveness of our new technique. Second, we carried out beach-based surveys to assess the social acceptance of this new surveillance approach, compared to a lethal shark mitigation strategy. Our results demonstrate blimp surveillance to be a promising and socially accepted tool for detecting sharks in proximity to ocean users. These findings challenge previous work^23^ that suggests aerial surveillance is limited in its application to shark mitigation.

“*It is better to be lucky. But I would rather be exact.*”

— Ernest Hemingway, The Old Man and the Sea

## Results

### Quantifying detection probability in a variable environment

**Figure 2:**
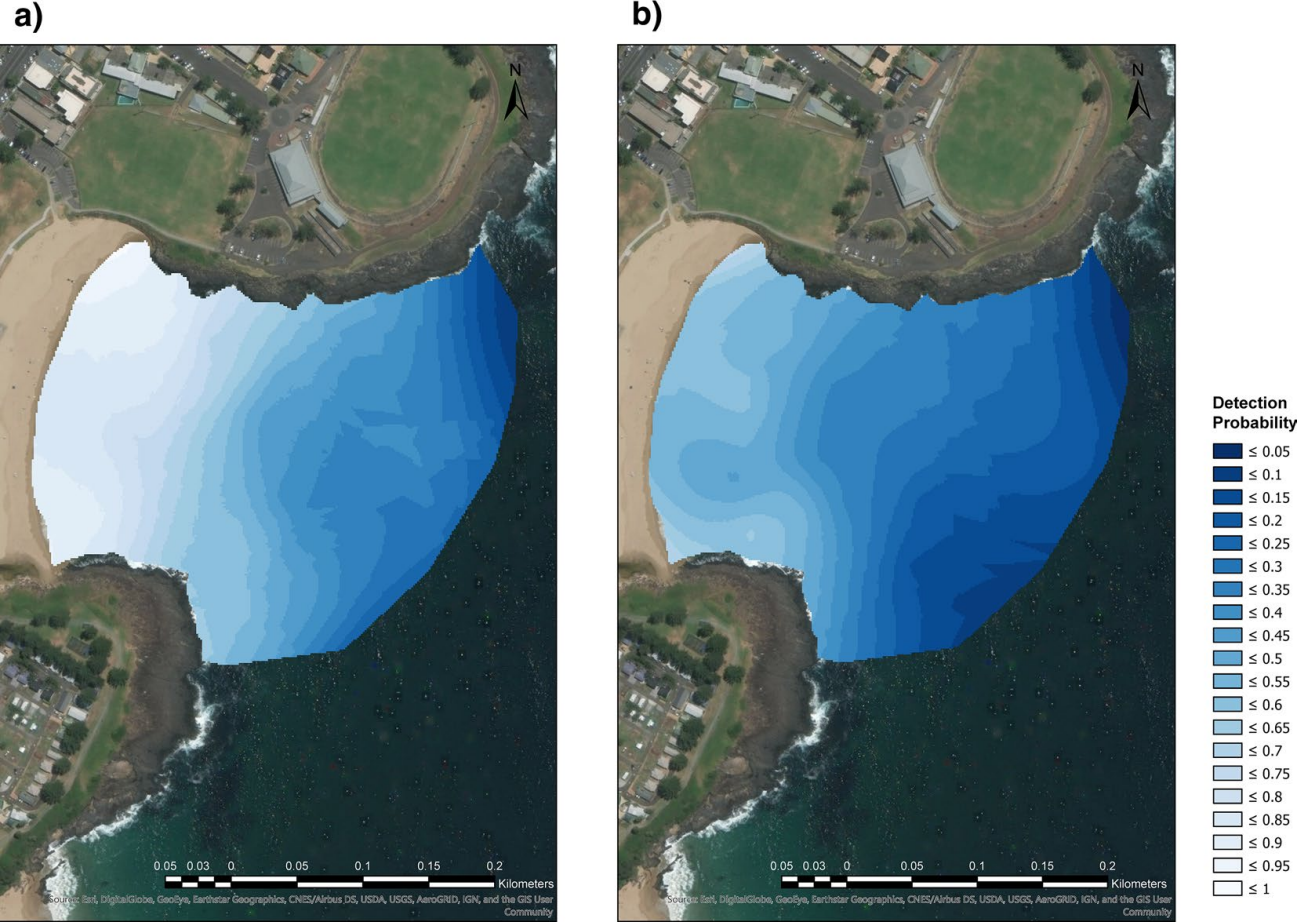
Shark analogue detection probabilities in (**a**) sunny (n = 29 analogue deployments over 5 days) and (**b**) cloudy (n = 22 analogue deployments over 5 days) conditions at Surf Beach in Kiama, NSW, Australia. Maps were created using ArcGIS Pro version 2.0.1 by Esri (www.esri.com).

The highest probability of lifeguards detecting shark analogues were in nearshore areas where swimmers usually frequent ocean beaches (Fig. 2). Detection probability decreased with increasing distance from shore until it approached zero. The probability of lifeguards detecting analogues in sunny conditions (Fig. 2a) was generally higher than in cloudy (Fig. 2b), and this was particularly apparent in the nearshore region (Fig. 2).

**Figure 3:**
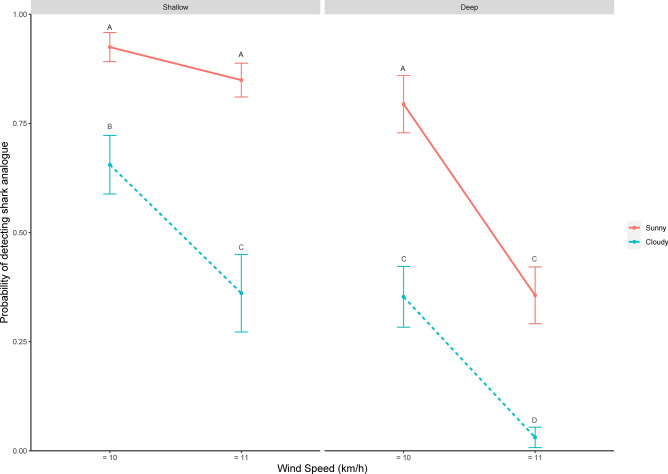
The probability of detection of 51 shark analogue deployments determined in trials by 20 lifeguards (± SE) in a double blind trial based on water depth (shallow: 2–3 m and deep: 4–5 m) versus cloud cover (sunny versus cloudy) and wind speed. Letters denote significant differences as determined by post hoc Tukeys multiple comparisons of means.

Understanding the environmental influences on shark detection probability is necessary to improve human safety. Shark analogue detection probability varied with environmental conditions (Table 1), but was extremely high (0.925 ± 0.0334) in ideal swimming conditions (i.e. sunny days and low winds) in areas where beachgoers tend to congregate (shallow water within the patrolled swimming area) and remained effective in stronger winds (0.849 ± 0.0387, Fig. 3; Table 1). With greater water depth, analogue detection on sunny days with low winds (0.794 ± 0.0657) was similar to that of shallow water with sunny days and higher winds (Fig. 3). Unsurprisingly, detection of analogues was reduced in shallow water with increasing cloud cover (0.655 ± 0.0671) and in windy conditions (0.361 ± 0.0889, Fig. 3). In stronger winds detection probability was lower (Fig. 3). In deep water, cloudy days with light winds had a detection probability of 0.353 (± 0.0696) and on sunny days with strong winds it was 0.356 (± 0.0650). Patterns in detection probabilities in deep water deployments generally mirrored those in shallow water but were reduced to a greater extent (Fig. 3). Detection in deep water and cloudy conditions with high winds was lowest and should be considered unreliable (0.0307 ± 0.0234, Fig. 3).

**Table 1:** The results from the generalized linear mixed model of the effect of environmental factors on shark analogue detection probability.

Fixed effects	Estimate	Standard error	z value	Pr ( >|z|)
Intercept	− 0.60732	0.30497	− 1.991	0.046435
Depth	1.24972	0.23997	5.208	< 0.000001
Sun	1.95722	0.35984	5.439	< 0.000001
Wind speed	− 2.84560	0.76339	− 3.728	0.000193
Depth: Sun	− 0.09162	0.53677	− 0.171	0.864467
Depth: Wind speed	1.63235	0.81739	1.997	0.045821
Sun: Wind speed	0.90366	0.82841	1.091	0.275347
Depth: Sun: Wind speed	− 0.47154	0.97688	− 0.483	0.629308

### Assessing Lifeguard Accuracy

**Figure 4:**
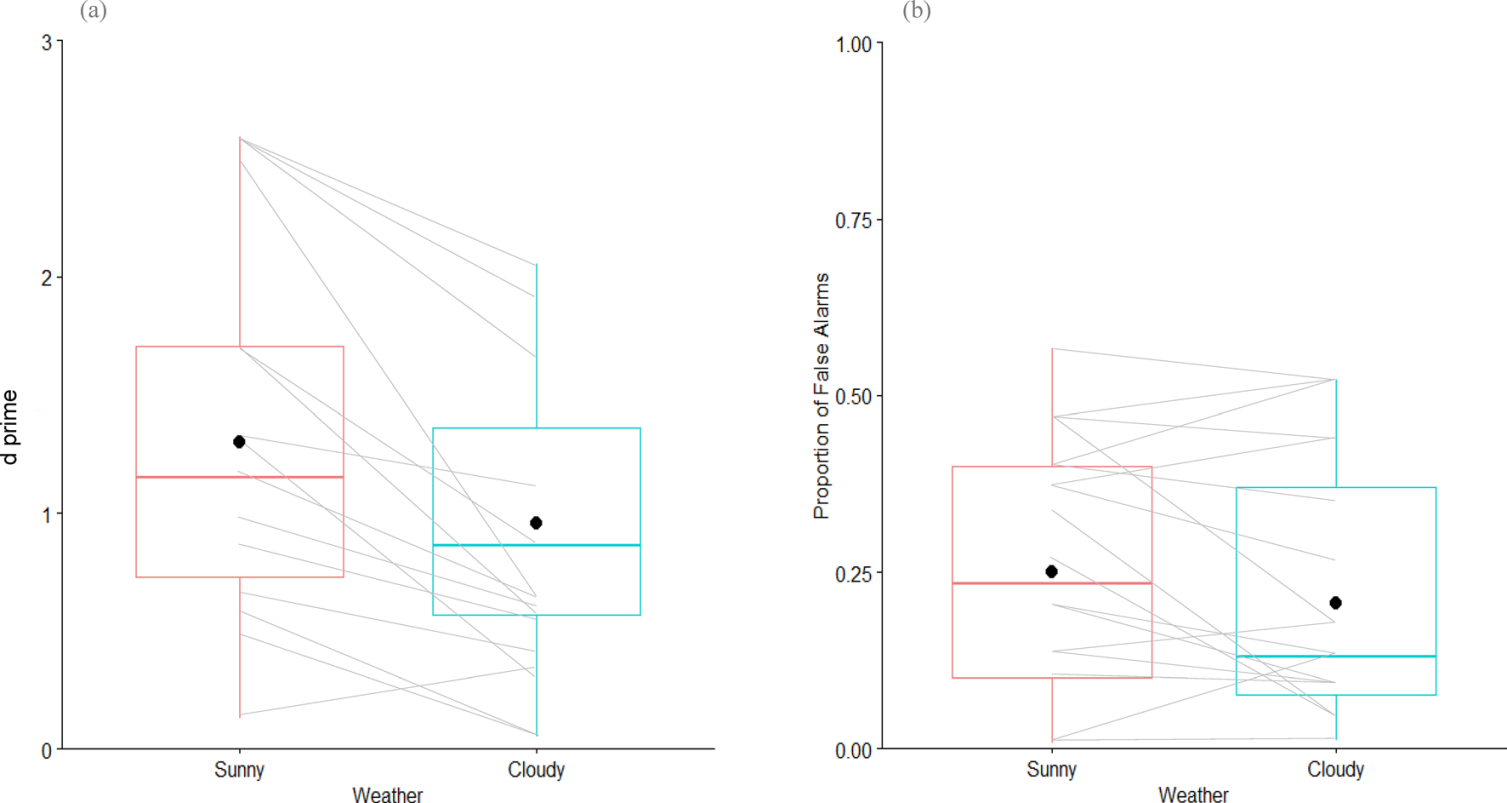
(**a**) Shark analogue detection sensitivity and (**b**) the proportion of false alarms of 20 professional lifeguards in Sunny and Cloudy conditions. Black point indicates the group mean. Grey lines join each individual lifeguard across the different weather conditions. A higher d prime indicates the easier discrimination of shark analogues from background noise (e.g. drift algae).

The human element (perception bias) of shark detection is important to quantify. Accuracy in this context is a measure of individual observer performance when using the novel technique and forms part of assessing the applicability of the whole shark-detection system. We quantified perception bias on two levels: sensitivity and false alarm rates. A higher detection sensitivity (dʹ) indicates easier visual discrimination of shark analogues from background ‘noise’ such as drift algae. The dʹ of lifeguards to detecting shark analogue presence was greater in sunny conditions than with cloud cover (t = − 2.83, df = 19, P = 0.01) (Fig. 4a). False alarms represent occasions when a lifeguard indicated they had seen a shark analogue when none was present, most likely resulting from observers spotting drift algae. False alarm rates were unaffected by cloud cover (t = − 1.71, df = 19, P = 0.10) and quite variable among lifeguards (Fig. 4b).

### Social acceptance

**Figure 5:**
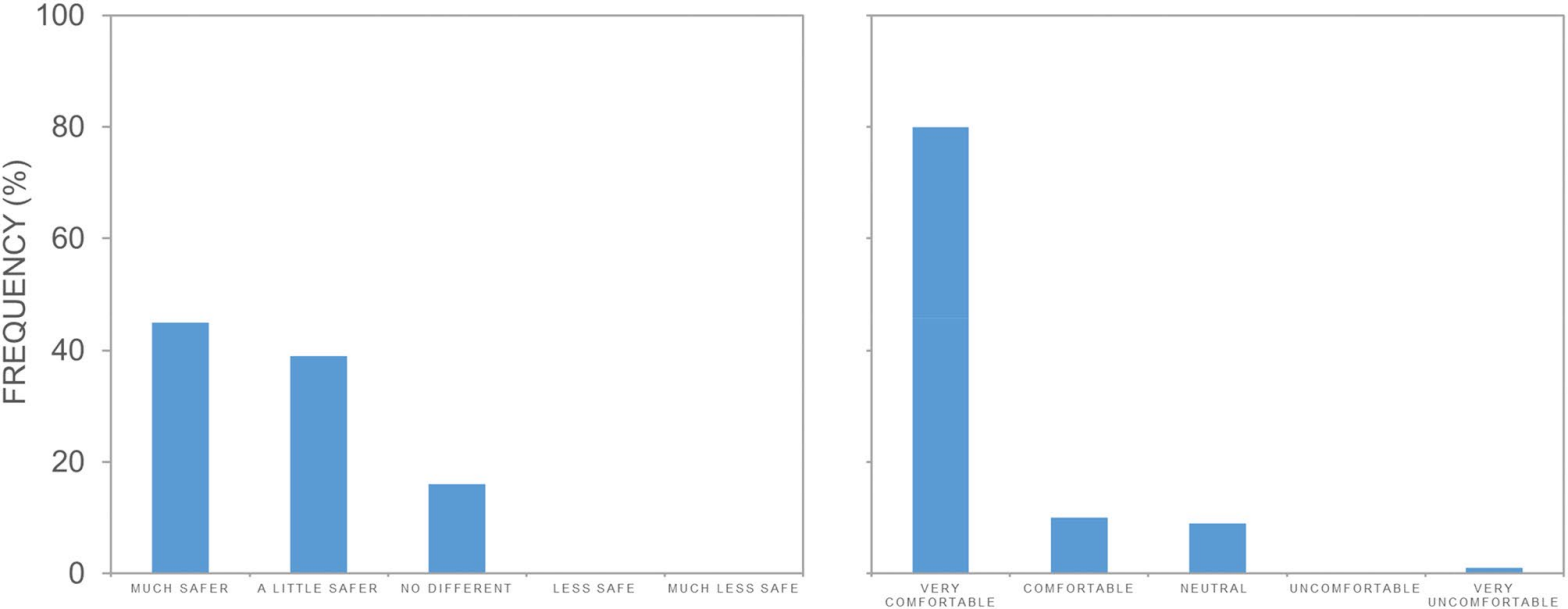
Frequency histogram showing responses to two related survey questions: (**a**) ‘Does the blimp give you a sense of safety from beach hazards?’ and (**b**) ‘Do you feel comfortable with the blimp at the beach?’.

Results of our survey completed by 115 beachgoers suggest wide acceptance of aerial surveillance by blimps. Beach activities varied among respondents, but the majority reported usually using the beach for recreation (71%), including swimming, body-boarding, body-surfing or playing in the breakers. Most respondents (74%) had entered the water on the day they completed the survey. On a five-point Likert scale, 84% of respondents said the blimp made them feel much safer (45%) or a little safer (39%); 16% reported feeling no different; none reported feeling less safe (Fig. 5a). Most respondents (90%) reported feeling very comfortable (80%) or fairly comfortable (10%) with blimp-based aerial surveillance at the beach; 9% were neutral; and 1% reported feeling very uncomfortable (Fig. 5b). The majority of respondents (67%) answered they would ‘choose to go to a beach with a blimp rather than one without, if both beaches were good and convenient’; 20% were undecided; and 13% reported they would not. Most (90%) stated they would like to see blimps at other beaches to improve beach safety; 10% were undecided.

**Figure 6:**
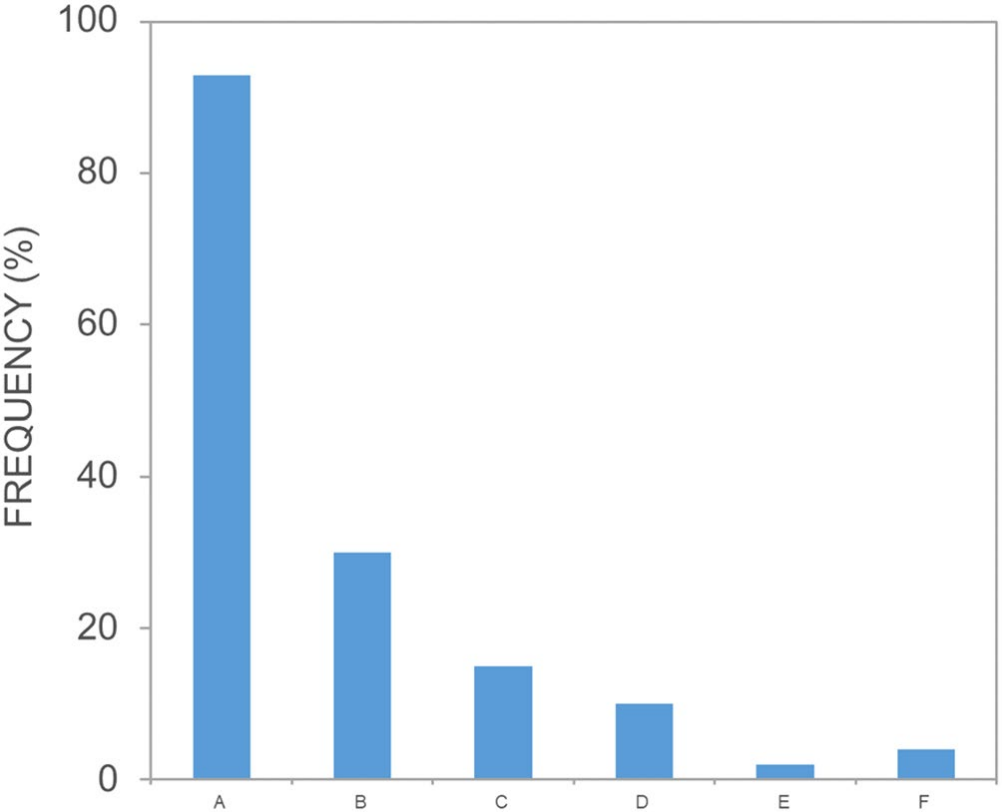
Frequency histogram showing responses to the survey question: ‘In general, what approaches would you like to see for keeping people safe from potential threats from sharks? (Choose as many options as you like)’ where A is ‘Spotting or detecting sharks, through methods that do not harm them’; B is ‘Relying on individuals taking responsibility for their own actions’; C is ‘Relying on personal deterrent devices, like electrical shields’; D is ‘Catching sharks and taking them off-shore, even if there is a risk of harming them’; E is ‘Catching and killing sharks’; and F is ‘Other’.

Respondents had mixed feelings regarding shark meshing. On a five-point Likert scale 39% had mixed feelings; 33% opposed (22%) or strongly opposed (11%); and 27% supported (18%) or strongly supported (9%) shark meshing. The sense of safety offered by shark meshing was mixed: 41% reported that it did not make them feel safer; 32% reported it did; 26% were undecided. Forty-five percent of people did not want to see shark meshing extended to cover the study area; 28% did and 27% were undecided. When asked to select from a list of five commonly adopted approaches they would like to see for keeping people safe from potential threats from sharks, the majority (93%) selected ‘Spotting or detecting sharks, through methods that do not harm them’. Least popular strategies were ‘Catching and killing sharks’ (2%) and ‘Catching sharks and taking them off-shore, even if there is a risk of harming them’ (10%) (Fig. 6).

## Discussion

“he knew no man was ever alone on the sea.”

— Ernest Hemingway, The Old Man and the Sea

Blimps can used as a tool to provide accurate, continuous and cost-effective aerial detection of sharks at beaches, offering lifeguards a unique and powerful tool for improving safety. Detection of shark analogues was most effective in shallow water (< 2 m)—the areas most commonly frequented by swimmers (Adams, pers. obs. 2018 (Adams has worked as a Professional Ocean Lifeguard for 9 years.)), and in sunny conditions—the time that beach visitation is highest^39,40^. In deeper water, where surfers are more common, the probability of detection remained high in good weather conditions. Higher wind speeds generally meant lower detection probability, which we attribute to two factors: increased camera movement and surface chop. When viewed collectively, this study highlights the practical application and limitations of this new technology for providing a measurable level of shark detection at ocean beaches.

Accuracy is key to the success of any novel technique to reduce potential interactions between sharks and bathers. Accuracy assessment has relevance to managers seeking to implement new technology, as well as observers themselves seeking to meet minimum performance standards. The high sensitivity of lifeguards to analogues deployed on sunny days provides further support for blimp-based aerial surveillance during popular beach use conditions. False alarms were most likely attributable to mobile drift algae, frequently observed to move slowly through the study area. In reality, a glimpse of this type would attract further scrutiny to confirm the observation. These trials were based on discrete events (20 s), while in a real-world scenario an object suspected of being a shark would be further observed to confirm identification. A shark is likely to be observed at several points in time as it moves through the area covered by the field of view of the blimp (250 m^2^). Typical cruising speeds of large coastal sharks are below 1 m per second^41^ providing observers’ sufficient time to view a shark in the “buffer zone” prior to it coming into direct contact with humans. The size of the buffer zone is likely to change depending on a number of factors, including cloud cover, wind speed and the beach profile. The adequacy of this buffer zone for providing an early warning system should therefore be assessed on a site by site basis.

The findings of our study are consistent with recent work that concluded drones were most effective for spotting sharks during light winds and in shallow clear water^42^. Depth below the surface is a key factor determining sightability of sub-surface objects from the air. Butcher and co-workers^42^ reported limited detection of shark analogues beyond 3.5 m using drones. The same is true for other aerial surveillance techniques. Robbins et al.^23^ and Westgate et al.^44^ also reported reduced detection ability at depth. These researchers concluded that the maximum deployment depth at which an analogue was detected was 4.3 m and 5 m when assessed by fixed-wing aircraft and helicopter respectively. A trial by Kelaher et al.^45^ compared manned (helicopter) to unmanned (drone) aircraft for detecting fauna and found drone pilots had lower precision. Drone pilots are required to fly the drone and spot fauna; therefore the difference in detection results was attributed to task loading. Notably, tethered blimps do not require a pilot, therefore the blimp observer would not experience this form of task loading. Of the surveillance methods considered, drones offer the most similar approach to blimps and have been shown to be effective monitoring tools for the detection of megafauna off coastal beaches^46^. Adams et al.^33^ offer a detailed comparison of the utility of a variety of aerial survey techniques and show the main limitations of blimp usage to be the need for storage of a fully inflated blimp, and the stability of the blimp in gusty or variable winds^33^. Blimps seem to be the most appropriate for fixed locations and long duration monitoring, whereas drones have greater mobility and can therefore cover larger areas of coastline, although for shorter time periods.

For shark monitoring, blimp-mounted cameras can outperform traditional methods of aerial surveillance, such as planes and helicopters, in several key areas. Importantly, the coverage and shark detection capacity they provide is continuous rather than restricted to the few minutes per beach per day for helicopter and fixed wing patrols. In terms of risk-reduction, the probability of detecting sharks from the blimp was high (93%) in ideal conditions. In comparison, fixed-wing aircraft and helicopter assessments achieved shark analogue spotting rates of 13% and 17% respectively^43^. The generally higher detection rates from the blimp are likely due to several factors. Robbins et al.^23^ deployed analogues at ~ 2 m below the surface in water 6–12 m deep on cloudy days. Detection rates of our shark analogue dropped in cloudy conditions and in deeper deployments to levels comparable with those found by Robbins et al.^23^. The blimp is also stationary, while the aircraft travel at over 30 m/s (110 km/h) making spotting a small target in the water difficult^47^. The shark analogues in Robbins et al.^23^ were stationary, while our analogues were moving, providing a more representative model of shark behaviour. The blimp was targeting shallow (2–5 m) near-shore swimming areas, which may increase the silhouetting of the analogues compared to the fixed-wing and helicopter assessments.

Shark mitigation demands balanced consideration of beach safety, species conservation^48^, and social acceptance. We found extremely high acceptance of blimps for beach safety, and strong preference for non-lethal strategies. In our study, acceptance comprised two elements: the blimp provided a sense of safety and respondents felt very comfortable in its presence. The majority of respondents stated they would choose a beach with a hazard mitigation blimp if all other factors were equal, and a large majority reported a desire to see blimps at more beaches to improve safety, suggesting that initiatives to introduce blimps would likely receive public support. In comparison, reported support for shark meshing was mixed, and much lower than for blimps. Few people reported that shark meshing made them feel safe, perhaps surprising given safety is the aim of meshing programs. It is important to note that while meshing is the main established technique for shark hazard mitigation in the state of New South Wales (in which the study was undertaken), the study site is not a meshed beach. Respondents may not have held detailed knowledge of how shark meshing operates. However, respondents reported very strong preference for methods that detect sharks without harming them. This suggests that people are accepting of non-lethal approaches to hazard mitigation; a finding consistent with other studies^6,22,27,48^. Indeed, shark mitigation strategy preference analysis by Simmons and Mehemet^22^ concluded that the likelihood of harm to sharks and other marine species is a central reason for community preferences. Several studies highlight the dilemma faced by managers tasked with mitigating dangerous shark incidents, and the reliance on established strategies that offer perception, but limited evidence, of risk reduction^6,10,49,50^. Respondents in our study were casual beachgoers who use the near-shore area for recreation. We believe this group represents the majority of beach-users and gives our findings strong relevance to managers and policy makers.

Our results indicate the potential for a reduction in the likelihood of a shark interaction for activities in the surf zone covered by the blimp, assuming people exit the water if a shark is sighted. Our technique is most applicable to activities held in a specific location (e.g. flagged swimming areas, surf contests, surf carnivals etc.). Surfing is one activity that has been suggested to offer more exposure to sharks^50^. Given the diffuse nature of this activity, the utility of our area-based approach would likely be limited to small beaches or specific sites such as organised surf competitions or singular surf spots. There will always be areas that cannot be patrolled due to remoteness and insufficient resourcing. Personal shark deterrents may offer an alternative strategy to reduce the likelihood of shark incidents for ocean-users outside patrolled hours or in remote locations but are also not 100% efficient in preventing a shark bite^50^. The blimp also has the proven ability to tap into current and emerging community-based shark alert systems that use acoustic telemetry or surveillance linked to smart-apps to warn of shark presence at beaches^38,51–55^. Blimp-based aerial surveillance shows promise as a highly visible, easily communicated and socially accepted shark hazard mitigation strategy.

Further development could be made with the use of multispectral cameras with blue light filters, which have been used previously to monitor sub-surface whales from space^56^. No doubt improvement could be made in this area using emerging remote sensing technologies, which are evolving rapidly. Turbidity has been shown to affect the detectability of fauna^42^ and therefore aerial surveillance for shark mitigation purposes is likely to be limited in highly turbid areas. The effect of time of day on spotting rates is particularly important to define, given the possibility of increased risk in shark incidents during dawn and dusk^3^. Time of day is also likely to impact the amount of surface glare on the water, which has been shown to impact the ability of observers to detect sub-surface objects^57,58^. It is important to note that beach visitation has been shown to peak during the middle of the day^36^ and many beaches are unpatrolled at dawn and dusk (Adams pers., obs. 2018). The results of our study demonstrate that blimp surveillance has direct and immediate application to global shark hazard mitigation and offers managers both a proactive strategy and a means of rapid response following a shark sighting or incident.

We conclude that blimps used as an aerial surveillance technique are demonstrably effective and represent a simple, cost-effective and socially accepted tool for mitigating the risk of shark incidents at ocean beaches. Lethal strategies have obvious environmental risks and ethical dilemmas^13,50^. Blimps may offer a non-lethal alternative with clear conservation benefits for those target and by-catch species caught in shark control programs. Our findings also have relevance to the potential effectiveness of other emerging shark surveillance methods such as drones^25,59^, which are currently limited by their short battery life. We envisage a role for expanding such research beyond shark hazard mitigation, to incorporate general beach safety and risk reduction, including from rips and other hazards. By providing an elevated platform for lifeguards (or algorithms) to monitor swimmers, blimps have further potential as a tool for reducing risk of ocean drownings; particularly important given occurrence of drowning deaths is an order of magnitude higher than shark incidents^22^. Our fusion of zeppelin-era technology with modern-day optics, communications and computing power provides a simple, environmentally sustainable and socially accepted method for improving beach safety, and could form part of a strategy that does not require lethal approaches to managing risks associated with human–shark encounter.

## Methods

### Study location

**Figure 7:**
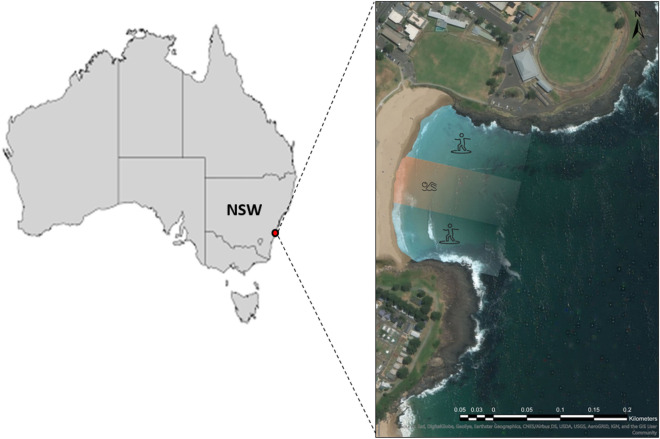
Surf Beach, Kiama is located on the south coast of NSW Australia. The beach is typical of a sandy coastal embayment and is enclosed by two fringing rocky reef headlands. Swimmers and surfers are frequent users of the bay over summer, with usage regulated by lifeguards. Swimmers are encouraged to swim within a flagged area where surfers are prohibited. Maps were created using ArcGIS Pro version 2.0.1 by Esri (www.esri.com).

The two phases of this study took place at Surf Beach, Kiama on the south coast of New South Wales (NSW), Australia (Fig. 7) during successive austral summers: December 2016–January 2017 for trials of the blimp and camera; and December 2017–January 2018 for social surveys. Surf Beach is within a sandy coastal embayment enclosed by two headlands (~ 250 m long; Fig. 7). Swimmers, surfers, and fishers are frequent users of Surf Beach over summer. The bathymetry of this area continually varies due to coastal processes, but generally water depth increases progressively to approximately 1.5 m depth at 50 m offshore. At this point there is a sudden depth increase to roughly 3 m and water depth then steadily increases to ~ 7–10 m around 300 m offshore (Adams pers. obs., 2018). Drift-algae primarily accumulate in the north end of the bay but can be found throughout the bay. Due to rip currents adjacent to each headland, bathers are encouraged to swim only in the central zone of the bay with lifeguards providing a supervised swimming area during summer (Adams pers. obs., 2018).

### Aerial platform and collection of imagery

We used a helium-filled blimp with a live streaming camera system to collect the imagery used in this study. The blimp design incorporates an 8,000 L helium-filled blimp and a gimbal-mounted camera (modified DJI Phantom 3 Advanced). Overall dimensions of the blimp were 5 m length and 1.8 m diameter with a payload of 2 kg. To minimise helium usage, the blimp was stored fully inflated with helium loss of less than 1% volume per day. The blimp was tethered above the beach at a height of 70 m, with camera settings fixed so they were consistent between trials (manual metering, ISO: 100 and Exposure: 100) (Fig. 1). Footage was displayed on a 24-inch monitor in the surf-club and recorded on a ground station (Atomos Ninja 2) at the transmitted 1280 × 720 p resolution with some image quality loss due to compression and through-air transmission. The position at which the blimp was situated depended on the wind direction and strength, and the anchor was thus placed at either the southern, central or northern areas of the beach as necessary. This minimised the offset of the blimp between trials and ensured the blimp could consistently observe the flagged swimming area.

### Shark analogue deployment

To establish a shark detection rate, and how it might vary with environmental conditions, we deployed a shark analogue on 10 days across a 6-week period encompassing a variety of weather and ocean conditions. The shark analogue was a moving free-diver of similar size to a juvenile great white shark (~ 2.5 m including dive fins). Using a waterproof GPS watch (Garmin Fenix 3) the free-diver logged the dive starting position and swam along the bottom in a straight line parallel to the shore for 20 s. Although the camera had the capacity to record to an on-board memory card, all footage used in this study was recorded on the ground station to ensure any image quality loss from transmission was incorporated into the detection estimate. Three shallow dives (2–3 m) and three deep dives (4–5 m) were conducted each day. We deemed a free-diver to be the most appropriate analogue for the surf zone which is a notoriously difficult area to deploy equipment and conduct research. The free-diver could safely navigate the surf, effectively simulate a moving animal and provide repeatable data. Water depth categories were sufficiently broad that they could be easily estimated by the experienced free-diver. Environmental variables were recorded during deployment, and included cloud cover, turbidity, wind speed and wave height. Wave height was estimated subjectively by the same observer at the time of analogue deployment. Cloudy conditions were characterised by the presence of clouds covering the sun during deployment. Wind speed was estimated from a weather station deployed at the study location and converted to a two-level categorical variable for analysis. Turbidity was consistent throughout the study period with the secchi depth never shallower than the deepest analogue deployment (5 m).

### Lifeguard shark analogue spotting trials

Footage collected during the trial was compiled and later shown to 20 professional lifeguards on a 24-inch HD monitor in a double-blind trial. Footage shown to lifeguards consisted of 104 randomised video clips (20 s duration); 51 having a shark analogue present and 53 showing the same beach with the shark analogue absent. The clips with the analogue absent were compiled from footage that was haphazardly selected from the 15 min prior or 15 min after the shark analogue was deployed each day to ensure minimal change in environmental conditions. Participants were asked to determine whether the shark analogue was present or absent, and to avoid subconscious prompting, the invigilator of the trial was not privy to the correct classification.

### Social surveys

Surveys were conducted to gauge public opinion of: (i) the novel use of a blimp-mounted camera; and (ii) approaches to shark hazard management more broadly. Questionnaire surveys were conducted face-to-face at the beach while the blimp was flying, to assess real-time attitudes towards the blimp. Questions focused on four elements: beach use; sense of safety and comfort with the blimp; general views on shark hazard mitigation; and views on the New South Wales *Shark Meshing (Bather Protection) Program*, which has been in operation since 1937. Broad demographic data were also collected. The beach surveys were conducted over three days in January 2018, during the NSW school holidays (peak beach usage) while the blimp was deployed. Kiama and the south coast are holiday destinations, so at this time local residents and tourists (predominantly from the state of NSW) visit the beach. Beachgoers were offered paper and electronic versions of the survey, which included a standardised briefing on shark meshing. Electronic versions were provided on tablets using the program SurveyMonkey. Beachgoers were approached directly by one of the researchers and asked if they were willing to participate in the research. To determine the level of public acceptance of aerial surveillance using a blimp, we investigated two measures: sense of safety and degree of comfort. Survey design and procedure were approved by the University of Wollongong Human Research Ethics Committee (HREC number 2016/993) and the survey questions are available as a supplementary file (supplementary material). All methods were carried out in accordance with relevant guidelines and regulations.

### Data analysis

We used two approaches to assess the performance of the blimp. First, we measured the average detection probability across lifeguards to assess external factor bias (known as availability bias) introduced by the environment, and second, we measured observer error (known as perception bias) by quantifying and comparing accuracy within and among lifeguards using signal detection theory.

### Shark analogue detection probability (availability bias)

To create a shark detection probability map that models the level of safety achieved at a beach by blimp surveillance, we used simple kriging to create two interpolated surfaces for sunny and cloudy conditions with analyses conducted in ArcGIS Pro version 2.0. Interpolation was based on the position of the sunny (n = 29) and cloudy (n = 22) shark analogue deployments and the proportion of each deployment that was spotted by the 20 Lifeguards. That is, if 20/20 lifeguards detected an analogue deployment, this point was assigned a value of 1 and, if 0/20 detected a deployment, that point was assigned a value of 0. To satisfy the assumptions of kriging in terms of error assessment, the data were arcsine transformed prior to interpolation. To make both maps comparable, the exploratory trend surface was standardised, and exponential polynomials were used to de-trend the data prior to fitting semi-variograms. In order to interpolate the risk-reduction map to cover the entire bay, two points were seeded at the high-water mark with a spotting value of one, and two points were seeded ~ 300 m offshore from the beach with a spotting value of zero. The interpolated surface was then truncated using the coastline as a barrier so that predictions only occur in water.

To compare the shark analogue detection probability under different environmental conditions we used a generalized linear mixed model fit by maximum likelihood (Laplace approximation) in the logit binomial family using the lme4 package in R^60,61^. Light conditions were included in the model as a two-level fixed factor (Sunny, Cloudy), and were crossed as a three-way interaction with water depth and wind speed, which were also two-level fixed factors; Shallow and Deep, and ≤ 10 km/h and 11–20 km/h respectively. Wave height was included as a random factor with 4 levels (1–4 feet) as we hypothesised that larger waves would have broader zones of white wash, making detection more difficult. Lifeguard was included in the model as another random factor to control for variation among observers. Significant interaction terms were further investigated using Tukey’s post-hoc multiple comparisons of means^62^ using the ‘multcomp’ package^63^.

### Assessing Lifeguard Accuracy (perception bias)

To assess the accuracy of the system we assessed the detection sensitivity, bias, and false alarm rate of lifeguards between two light conditions (Sunny vs Cloudy) using signal detection theory^64^. A yes/no trial in signal detection theory results in one of four possible outcomes (Table 2).

**Table 2:** Signal detection outcomes based on the presence or absence of shark analogues, visual ‘noise’ and the response of lifeguards.

Shark analogue present				Lifeguard response		Outcome
Yes	+	Noise	+	Positive	=	Detection
Yes	+	Noise	+	Negative	=	Miss
No	+	Noise	+	Positive	=	False alarm
No	+	Noise	+	Negative	=	Correct rejection

Sensitivity to stimuli (dʹ) provides a summary of the ability of lifeguards to distinguish shark analogues (signals) from ‘visual’ noise^64^. The higher the value of dʹ the more sensitive a lifeguard is to stimuli^64^. Signal detection theory metrics were calculated for each lifeguard in sunny conditions and cloudy conditions. A standard correction was applied when the hit or false-alarm rate equalled 0 or 1^64,65^. Depth was unable to be included in these analyses, as no value for water depth could be assigned when the shark analogue was absent. The average values of sensitivity (dʹ) and bias (C) were compared using paired t-tests to account for inherent differences in individual lifeguards.

## Supplementary information

Supplementary file1
