# Supplementary material for: Coexisting with sharks: a novel, socially acceptable and non-lethal shark mitigation approach
Source: Sci Rep. 2020 Oct 15;10:17497. doi: 10.1038/s41598-020-74270-y (PMC7562904; doi:10.1038/s41598-020-74270-y)
Supplement: Supplementary file 1 — Supplementary file1 [file 41598_2020_74270_MOESM1_ESM.pdf]

# Project AIRSHIP
# Shark spotting trial

**Project AIRSHIP** is a low-cost shark spotting program that provides continuous coverage of an area such as a swimming zone or surf bank, via a blimp.

We are researchers from the University of Wollongong currently testing the effectiveness of this technique.

We are interested in beach-goers' views about Project AIRSHIP, and other shark hazard mitigation techniques. We would like to hear from you—as a beach-goer—about your views.

We have designed a survey that we hope you'll fill out. It should take10-15 minutes to complete. Your responses will be anonymous, and you can decide to withdraw at any time.

We plan to publish our results in academic journals and public forums, and present them at relevant conferences. We hope they will be used to inform public debate and policy.

If you have questions about the survey, or our research, you can contact us by email: Dr Leah Gibbs (leah@uow.edu.au) or Kye Adams (ka738@uowmail.edu.au). If you have concerns about the way the research is conducted, you should contact the University Ethics Officer on 02 4221 4457 or rso-ethics@uow.edu.au.

Thank you very much for contributing to our research.

---

## Your beach use

1. What day of the week are you visiting the beach today? ___________________

2. During the warmer months, how often do you visit the beach? ___________________

3. Who do you usually visit the beach with?

☐ I usually come alone
☐ With my family
☐ With a friend(s) or partner/spouse
☐ With a club or team
☐ With my children
Other (please specify) ___________________

4. How would you describe your level of confidence in the water at the beach?

☐ Very confident
☐ Fairly confident
☐ Neither confident nor unconfident
☐ Fairly unconfident
☐ Very unconfident

5. What do you usually do at the beach? (choose all that apply)

☐ Sit, walk or exercise on the sand
☐ Wade or play in shallow water
☐ Swim, body-board, body-surf, play in breakers
☐ Swim behind the breakers
☐ Surf
☐ Snorkel
☐ Dive
☐ Fish from the shore, rocks or boat
☐ Fish from the water (e.g. spear-fish)
Other (please specify) ___________________

## Your views about Project AIRSHIP

6. Have you noticed the Project AIRSHIP blimp today? ☐ Yes ☐ No

7. Did you swim or enter the water at the beach today? ☐ Yes ☐ No

8. In a few words, how did it feel to be in the water beneath the blimp? Or, if you didn't enter the water, how does it feel to be at the beach with the blimp?

9. Does the blimp give you a sense of safety from beach hazards?

☐ It makes me feel much safer ☐ It makes me feel a little safer

☐ It doesn't make me feel anything or any different

☐ It makes me feel a little less safe ☐ It makes me feel much less safe

10. Do you feel comfortable with the blimp at the beach?

☐ I feel very comfortable ☐ I feel fairly comfortable

☐ I feel neither comfortable nor uncomfortable

☐ I feel a little uncomfortable ☐ I feel very uncomfortable

11. Would you choose to go to a beach with a blimp rather than one without, if both beaches were good and convenient? ☐ Yes ☐ No ☐ Undecided

12. Would you go out of your way to visit a beach with a blimp? ☐ Yes ☐ No ☐ Undecided

13. Would you like to see blimps at other beaches to improve general beach safety?

☐ Yes ☐ No ☐ Undecided

## Your views about other strategies

14. Have you heard of the NSW Shark Meshing Program, sometimes referred to as 'shark nets'?

☐ Yes ☐ No

If 'no' go to question 24.

## NSW Shark Meshing Program

Thinking about the NSW Shark Meshing Program, please answer the following questions to the best of your knowledge.

15. When are the nets set?

☐ All year round

☐ In the warmer months

☐ In the cooler months

☐ I don’t know

Other (please specify) ___________________

16. Where are the nets set?

☐ All NSW beaches

☐ All beaches between Newcastle and Wollongong

☐ All Sydney beaches

☐ Some beaches between Newcastle and Wollongong

☐ I don’t know

Other (please specify) ___________________

| | | | |
|---|---|---|---|
| 17. Are the nets visible from the beach? | ☐ Yes | ☐ No | ☐ I don't know |
| 18. Nets are designed to create an enclosed area for swimmers. | ☐ Yes | ☐ No | ☐ I don't know |
| 19. Sharks can swim around or over nets. | ☐ Yes | ☐ No | ☐ I don't know |
| 20. Nets are designed to catch sharks. | ☐ Yes | ☐ No | ☐ I don't know |
| 21. Nets catch other animals as well as sharks. | ☐ Yes | ☐ No | ☐ I don't know |
| 22. Most animals caught are potentially dangerous to people. | ☐ Yes | ☐ No | ☐ I don't know |
| 23. Most animals caught in shark nets are released unharmed. | ☐ Yes | ☐ No | ☐ I don't know |

—

24. The NSW Shark Meshing Program was introduced in 1937. It involves fishing nets being set in the water from November to April each year at some beaches between Newcastle and Wollongong. Kiama beaches are not part of the program.

The fishing nets are set about 500m off shore. They do not create a barrier. They are 150m long and 6m deep, set at beaches much longer than 150m in water up to 12m deep. This means that sharks and other animals can swim around and over the nets.

The Shark Meshing Program works by catching sharks that might pose a threat to beach-goers. Sharks and other animals are caught and some are killed by the nets.

How do you feel about the NSW Shark Meshing Program?

☐ I strongly support it

☐ I support it

☐ I neither support nor oppose it, or I have mixed feelings about it

☐ I oppose it

☐ I strongly oppose it

| | | | |
|---|---|---|---|
| 25. Does the Shark Meshing Program make you feel safe? | ☐ Yes | ☐ No | ☐ Undecided |
| 26. Would you like to see the Shark Meshing Program in Kiama? | ☐ Yes | ☐ No | ☐ Undecided |

## Your views of shark hazard management

27. Do you think some form of shark hazard management should be in place at NSW beaches?

☐ Yes ☐ No ☐ Undecided

28. In general, what approaches would you like to see for keeping people safe from potential threats from sharks? (Choose as many options as you like)

☐ Catching and killing sharks

☐ Catching sharks and taking them off-shore, even if there is a risk of harming them

☐ Spotting or detecting sharks, through methods that do not harm them

☐ Relying on personal deterrent devices, like electrical shields

☐ Relying on individuals taking responsibility for their own actions

Other (please specify) ________________

29. Who do you think is responsible for keeping people safe from sharks? (Choose all that apply)

☐ State and/or federal government

☐ Private corporations

☐ Everyone at the beach

☐ Each individual

☐ Local councils

☐ Lifeguards and Lifesavers

☐ Family and friends of beach-goers

Other (please specify) ________________

## About you

To understand patterns in our survey data it helps us to know a few things about you.

30. What is your gender? ☐ Woman ☐ Man ☐ Prefer not to say, or other

31. What is your age or age group (choose a five-year band; e.g. 30–34; 65–69)? ________________

32. In what country do you live? ☐ Australia ☐ Other (please specify) ________________

33. What is your postcode? ________________

34. In what country were you born? ☐ Australia ☐ Other (please specify) ________________

35. Where have you lived most of your life? ☐ Australia ☐ Other (please specify) ________________

## Other comments

36. Do you have any other comments you would like to share with us about Project AIRSHIP or shark hazard mitigation?

**Thank you for contributing to our research.**
